# Proton wave propagation along collagen fibers

**DOI:** 10.1007/s00249-026-01856-1

**Published:** 2026-07-29

**Authors:** Pedro Costa, Anna Maznichenko, Noam Agmon, Peter Pohl

**Affiliations:** 1 Institute of Biophysics, Johannes Kepler University Linz, Linz, 4020 Austria; 2 Institute of Chemistry, The Hebrew University of Jerusalem, Jerusalem, 9190401 Israel

**Keywords:** Interfacial water, Extracellular matrix, Tumor invasion, Fluorescence microscopy, Grotthuss mechanism

## Abstract

Collagen deposition and alignment in the tumor stroma stiffen the extracellular matrix and, via integrin signaling, stimulate proton extrusion by cancer cells. In aggressive tumors, migration occurs preferentially along aligned collagen fibers, where proton efflux at the leading edge acidifies and degrades the matrix to facilitate invasion. Whether directional proton diffusion along collagen fibers exacerbates invasion remains uncertain, as it is not known how far protons can travel along a fiber before being released. To characterize the release barrier, we labeled collagen lysine side chains with a pH-sensitive fluorescein derivative and introduced an interfacial proton flux by microinjecting HCl near the surface of self-assembled collagen fibers elevated above the chamber bottom. Proton wave propagation was monitored by wide-field fluorescence microscopy. Quantitative analysis of the fluorescence transients revealed that proton migration is best described by a one-dimensional diffusion model with a finite surface-to-bulk release rate. A single global parameter set consistently described all experiments. From the fitted release rate, we determined a Gibbs activation energy barrier Δ*G*^‡^ of approximately 30 *k*_B_*T*, comparable to values reported for membrane–water interfaces. These results demonstrate that protons can travel tens of micrometers along collagen fibers before equilibrating with the bulk solution, suggesting that membrane-anchored collagen fibers may provide an effective pathway for directing acidification along migration tracks in tumor stroma.

## Introduction

Collagen is the most abundant protein in the human body, forming the principal structural component of the extracellular matrix in various connective tissues, including skin, bone, cartilage, and tendons (Gelse et al. 2003; Ricard-Blum 2011). Its triple-helical structure provides mechanical strength and is essential for maintaining tissue integrity and function (Kadler et al. 2007; Shoulders and Raines 2009). Alterations in extracellular pH have profound effects on cancer cell behavior, tumor progression, and response to therapy (Ward et al. 2020).

Increased collagen deposition and fiber alignment in the tumor stroma stiffen the extracellular matrix (ECM), which - through integrin signalling - leads to increased acid extrusion by cancer cells (Bourgot et al. 2020; Fang et al. 2014). Tumor cells migrate along collagen tracks - regions where proton-extruding machinery concentrates at the leading edge, promoting movement by acidifying and digesting adjacent ECM. Consequently, highly aligned collagen is associated with increased cell migration and a more aggressive phenotype (Bourgot et al. 2020; Carey et al. 2012; Winkler et al. 2020).

Interestingly, biomimetic studies implicated collagen in facilitating proton transport (Matsuo et al. 2017; Palsaniya et al. 2025; Semizo et al. 2023), prompting the question of whether this property contributes to tumor spread. To date, however, nearly all experimental studies—including proton conduction measurements and applications such as fuel cell membranes—have been performed in humid air or under controlled humidity, not with collagen submerged in liquid water (Matsui and Matsuo 2020; Matsuo et al. 2017; Shibata et al. 2025). Collagen lacks proton conductivity when dry, as a network of water molecules integrated into the structure and connecting peptide chains via hydrogen bonds is necessary (Matsui and Matsuo 2020). Conductive properties strongly depend on relative humidity; proton transport becomes efficient only above 80% humidity (Matsui and Matsuo 2020; Matsuo et al. 2017). Impedance studies confirm that conductivity is regulated by rearrangement of hydrogen bonds (Matsui and Matsuo 2020).

Amino acids like hydroxyproline play a key role in proton transport across collagen fibers by extending the hydrogen bond network (Orieshyna et al. 2023). The latter makes hydrated collagen an appealing material for biocompatible proton exchange membranes in fuel cells, showing effective proton transport when adequately hydrated and achieving notable power densities under moist (not wet) conditions (Tawalbeh et al. 2025; Palsaniya et al. 2025).

The observation that protons travel along collagen-supported, hydrogen-bonded networks raises the question: Does the proton conductivity of such a network provide directionality to proton diffusion compared to random diffusion in solution? This phenomenon could support a mechanism where protons propagate preferentially along interfaces (Comtet et al. 2020; Serowy et al. 2003; Sjoholm et al. 2017; Toth et al. 2020; Zhang et al. 2012). A similar mechanism has been proposed in mitochondria, where interfacial proton diffusion between respiratory proton pumps and ATP synthase is thought to enhance energy conversion efficiency. Conversely, under conditions of elevated electrochemical potential, protons may bypass ATP synthase and travel laterally along the membrane to more distant uncoupling proteins, which dissipate the potential by facilitating transmembrane proton transport (Klotzsch et al. 2015).

Whether collagen fibers can facilitate proton movement in the direction of tumor invasion depends on how far protons travel along the interface before release into the surrounding extracellular aqueous phase—a process likely hindered by an energy barrier, as known for lipid bilayers and biological membranes (Antonenko et al. 1993; Heberle et al. 1994; Knyazev et al. 2023; Weichselbaum et al. 2017). However, energetics of the retention and release of protons from collagen surfaces into bulk solution remain unclear.

**Fig. 1:**
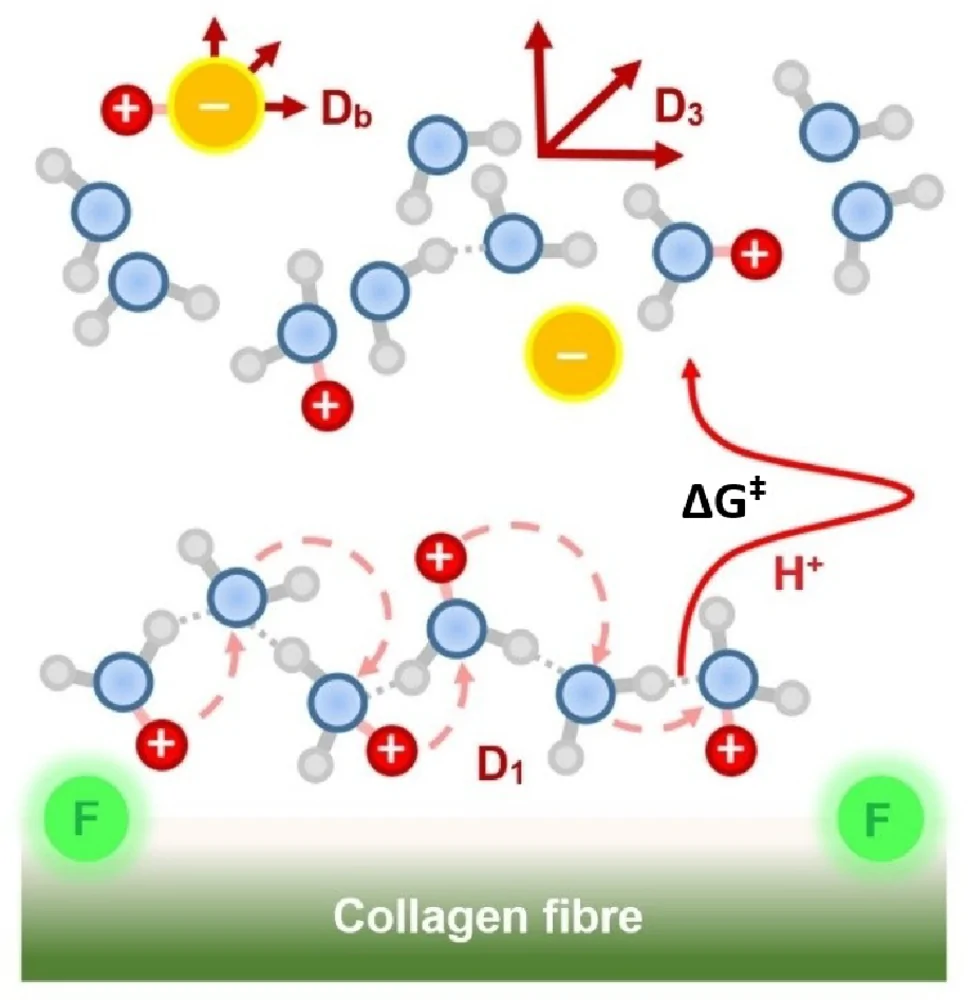
Model of proton transport along collagen fibers. Fast one-dimensional (1D) proton surface diffusion (*D*₁) occurs along structured interfacial water adjacent to collagen fibers via the Grotthuss mechanism. To irreversibly escape into the bulk phase, protons must overcome a Gibbs activation energy barrier Δ*G*^‡^. Bulk proton diffusion occurs in unstructured water and constitutes a three-dimensional (3D) process. In buffered solution, protons are predominantly carried by buffer molecules with diffusion coefficient *D*_b_; only under conditions of buffer depletion may contributions from hydronium diffusion or proton self-diffusion (*D*₃) become relevant

Using collagen-anchored fluorescent pH sensors, we set out to measure the migration distance of protons along collagen structures and determine the proton release barrier. We assess whether proton movement along collagen fibers can be described by a one-dimensional diffusion model, and quantify the Gibbs activation energy Δ*G*^‡^ for proton escape from the surface (Fig. 1).

## Materials and methods

Collagen fibers were formed by self-assembly in aqueous solution. Stock bovine collagen type I was prepared from fibrous powder according to the manufacturer’s instructions (Sigma‑Aldrich, collagen from bovine flexor tendon, Cat. No. 5162) with minor modifications. First, we dissolved 1 g of collagen powder in 50 ml of cold distilled water. The mixture was continuously stirred for 15 min until the collagen was fully hydrated. Subsequently, 50 ml of cold 0.02 M HCl was added, and the solution stirred for 10 min, resulting in a final collagen concentration of 10 mg/ml. The pH was verified to fall between 2 and 3. Afterwards, the mixture was further homogenized on a stir plate for one hour. The temperature was maintained slightly over 24 °C throughout. The stock solution was stored at 4 °C until use.

Fiber assembly followed routine collagen coating guides (see ThermoFisher Rat Tail Type I Collagen, Cat. No. A1048301). The stock solution was diluted to 50 µg/ml in 0.02 M acetic acid. The diluted collagen was applied to a borosilicate glass coverslip (0.15 mm thickness) to ensure 5 µg of collagen per cm². The samples were then incubated for one hour at 37 °C. Excess solution was subsequently removed, and the coverslips were rinsed three times with a buffer containing 0.1 mM 3-(cyclohexylamino)-2-hydroxy-1-propanesulfonic acid (CAPSO) and 50 mM NaCl.

Collagen labeling was achieved by adding 500 µL of fluorescein activated as its *N*-hydroxysuccinimide ester, dissolved in dimethyl sulfoxide (7 µg mL⁻¹), to the collagen fibers on coverslips. Under these conditions, the dye reacted covalently with primary amines, predominantly lysine side chains of collagen. Coverslips were incubated at room temperature for 1.5 h in the dark to prevent photodegradation of the fluorophore. Unreacted fluorescein was subsequently removed by washing the coverslips three times with the aforementioned CAPSO buffer.

**Fig. 2:**
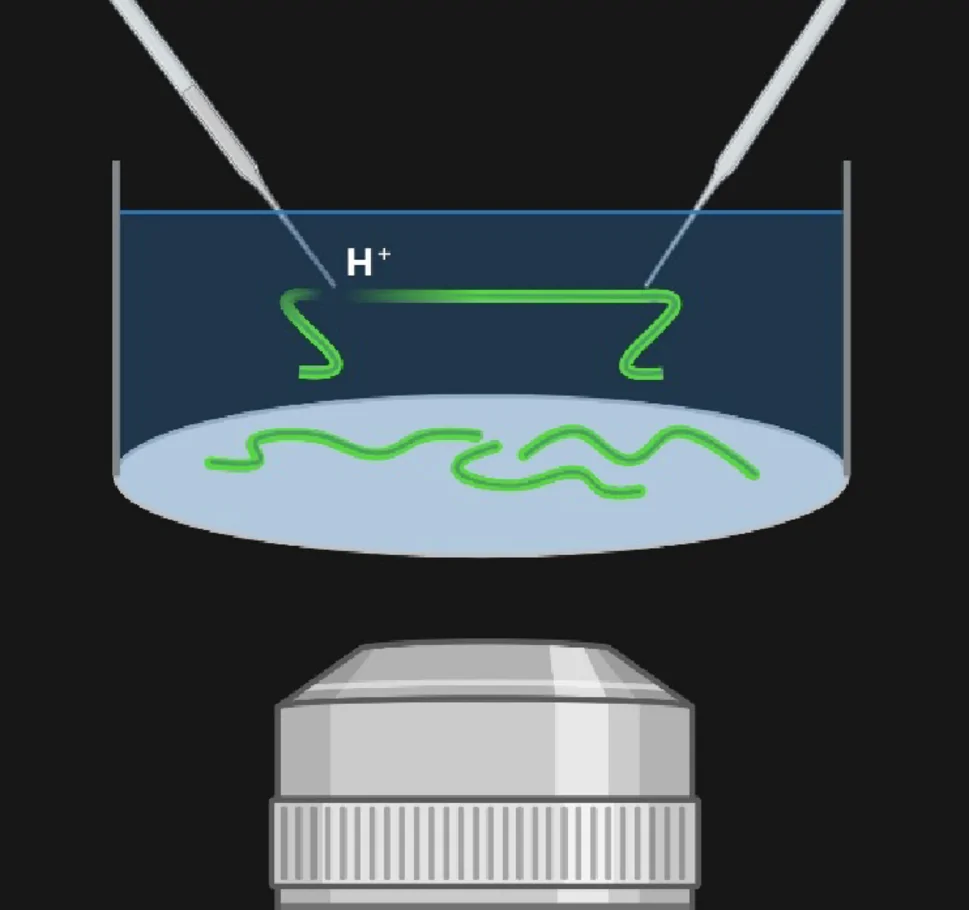
Schematic illustration of the experimental design. The left micropipette delivers HCl, initiating a proton wave that propagates along the submerged collagen fiber and transiently quenches the fluorescence of surface-bound pH sensors. Images were acquired with a time resolution of ~ 0.02 s, covering the full evolution of the proton wave from injection to recovery of the initial surface pH

For fluorescence measurements, the CAPSO buffer was gently added onto the coverslips to submerge the collagen fibers. Although the bulk solution was adjusted to pH 9.0, local proton microinjection transiently reduced the surface pH into the range of approximately 7–8, overlapping with physiological conditions. The elevated bulk pH was chosen to enhance the fluorescence sensitivity of surface-bound fluorescein, whose apparent pKₐ increases upon immobilization, while NaCl was included to screen excessive electrostatic interactions. Selected fibers were lifted tens of micrometers above the surface using two micropipettes (Fig. 2). Proton microinjection was performed via the left pipette (Antonenko and Pohl 2008; Zhang et al. 2012). Coverslips were placed over a wide-field microscope objective. Covalently attached pH sensors (fluorescein-labeled hydroxylysine side chains) were excited with a 488 nm laser to visualize proton wave propagation.

## Results

**Fig. 3:**
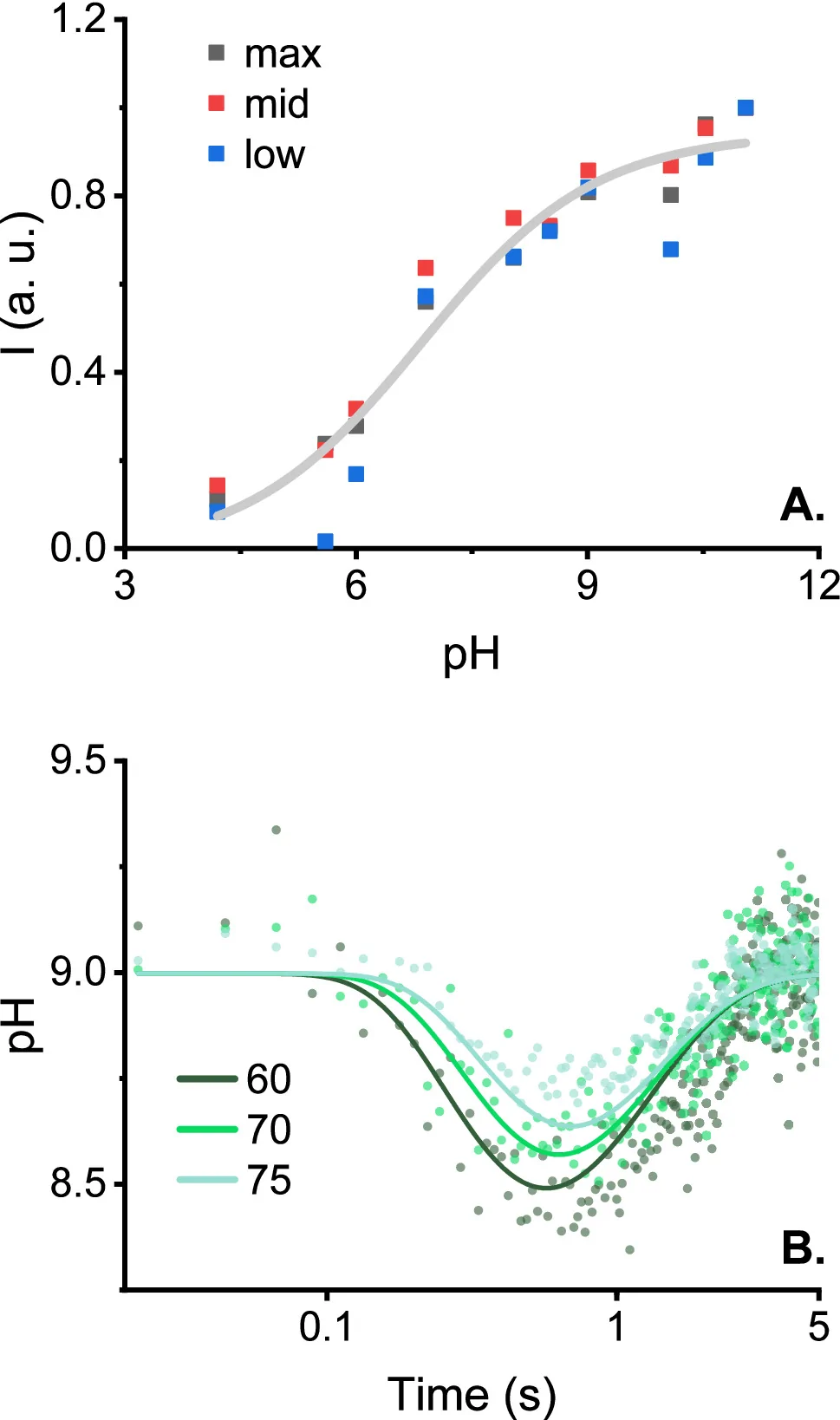
pH changes induced by proton wave propagation along collagen fibers. (***A***) Calibration curve relating the relative fluorescence intensity of collagen-anchored fluorescein (normalized to its value at pH 11). Fluorescence intensities were measured at fiber regions with high, intermediate, and low fluorophore density. (***B***) Representative fluorescence time traces recorded at three distances, *d*, from the proton release site, illustrating the transient decrease and subsequent recovery of fluorescence intensity as the proton wave passes. Because the relative change in fluorescence intensity was independent of the density of the fluorescent label, we performed a global fit of the analytical model (Eq. 1) to all traces (solid lines). This analysis yielded an effective one-dimensional diffusion coefficient of *D*₁ = 1332 ± 96 µm^2^s^-1^, and the release constant *k*_off_ = 1.2 ± 0.2 s^-1^

To quantify local pH changes arising from proton wave propagation along the fiber, the pH-dependent fluorescence response was calibrated. The fluorescence intensity measured at pH 11 was normalized to unity, and relative intensity changes following acid addition were subsequently recorded (Fig. 3A). The resulting calibration curve closely resembled those obtained previously for the same pH-sensitive dye anchored in lipid bilayers.

Microinjection of protons at a defined position along the fiber generated a proton wave that propagated along its length. Upon reaching a distant site, the fluorescence intensity recorded at that location decreased and subsequently recovered to its initial value, indicating passage of the wave (Fig. 3B).

Three representative fluorescence transients recorded from a single fiber at three different distances *d* from the proton injection point are shown. The time *t*_max_ at which the fluorescence intensity reached its minimum—corresponding to the maximal local deviation of pH from its baseline value—increased systematically with increasing distance *d*. To guide the eye, smooth curves representing fits of an analytical model to the data are superimposed (Fig. 3B).

The model describes one-dimensional diffusion combined with an exponential decay term, accounting for proton release from the fiber surface (Agmon and Gutman 2011):

1 $$\:c\left(x,t\right)={c}_{0}+\frac{A}{(4{\uppi\:}{{D}_{1}t)}^{\frac{1}{2}}}\mathrm{exp}\left(\frac{-{x}^{2}}{4{D}_{1}t}\right)\mathrm{exp}\left(-t\:{k}_{\mathrm{o}\mathrm{f}\mathrm{f}}\right)$$

where *c*_0_ is the initial proton concentration, *A* is a scaling factor related to the transient proton concentration increase induced by microinjection, *D*_1_ is the effective one-dimensional diffusion coefficient along the fiber, *x* is the proton travel distance and *k*_off_ denotes the first-order rate constant describing proton dissociation from the fiber surface into the surrounding bulk solution.

**Fig. 4:**
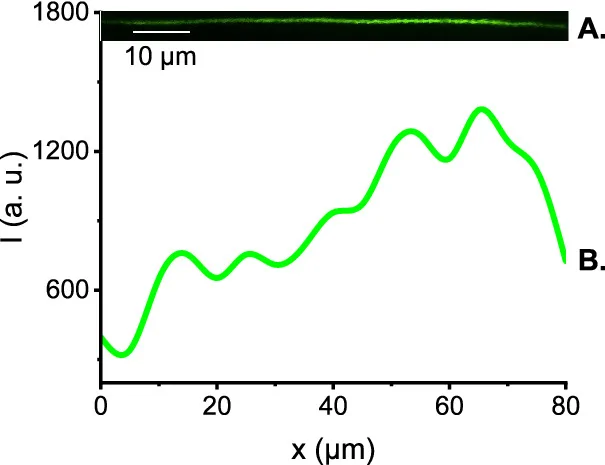
Fluorescence microscopy of a collagen fiber labeled with fluorescein. (**A**) Representative fluorescence image of a collagen fiber labeled with the N-hydroxysuccinimide ester of fluorescein. The image was acquired after removal of excess, unreacted dye by washing, leaving fluorescein bound to the fiber. (**B**) Fluorescence intensity profile measured along the fiber prior to proton microinjection, revealing an inhomogeneous distribution of fluorophore density as a function of distance from the injection site

However, because fluorescence intensity was not homogeneous along the fiber (Fig. 4A and B), it was not possible to calculate the diffusion constant from a single snapshot.

Thus, we calculated the one-dimensional diffusion constant *D*₁ of protons on the surface of a collagen fiber from fluorescence intensity time traces measured at positions distant from the release site. Two approaches were applied:

**First**, we exploited the fact that these traces (Fig. 3B) exhibit a clear minimum corresponding to the maximum of proton concentration. Denoting the time* t* at which this maximum was reached as *t*_max_ and the corresponding distance *x* from the proton release site as *d*, we plotted *t*_max_ as a function of *d*^*2*^ to determine 𝐷_1_. The one-dimensional diffusion equation predicts a strictly linear function:

2 $$\:{d}^{2}=2\:{D}_{1}{t}_{\mathrm{m}\mathrm{a}\mathrm{x}}$$

**Fig. 5:**
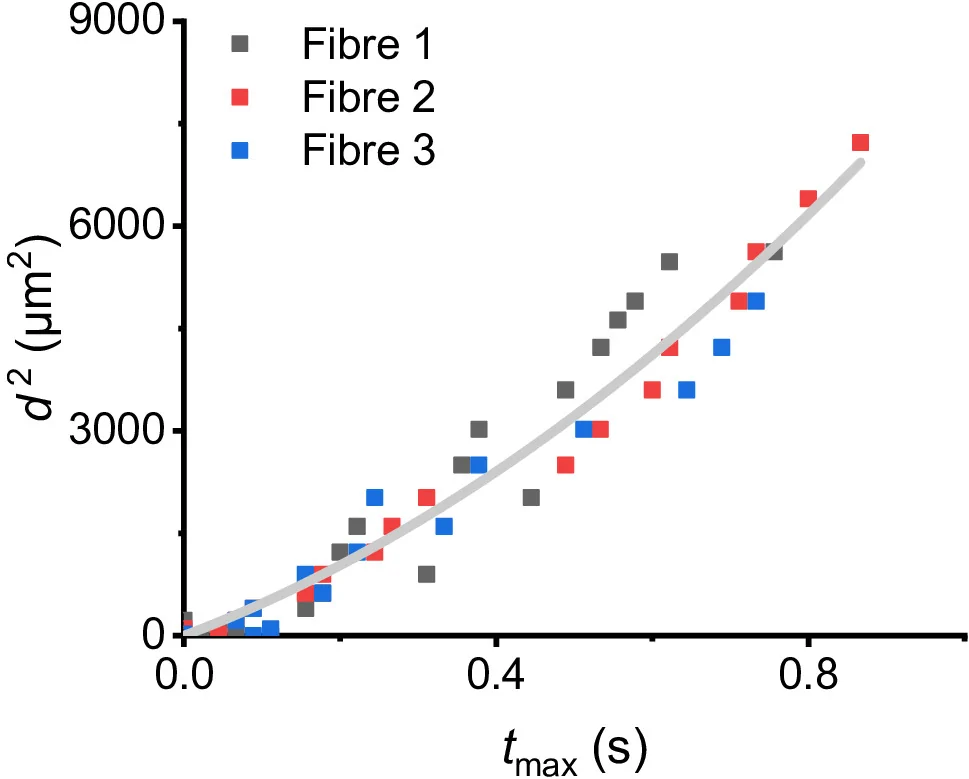
Distance dependence of proton propagation along collagen fibers. The time t_max_ at which the fluorescence reached its minimum (corresponding to maximal proton concentration) increased with distance from the injection site. Plotting *t*_max_ versus squared distance revealed a deviation from strict linearity, indicating finite proton release from the fiber surface. Fitting Eq. (3) yielded a one-dimensional surface diffusion constant *D*₁ = (2153 ± 290) µm² s⁻¹ and a proton release rate *k*_off_ = 0.5 ± 0.2 s⁻¹. Data from three independent fibers are shown

Yet, we found a slightly bent shape (Fig. 5), indicating that a correction factor for the proton release from the fiber to the bulk (via a rate coefficient *k*_off_) must be included. We derived *k*_off_ using Eq. 1. Since at *x* = *d* the time derivative of *c(x*,* t)* vanishes, we obtain:

3 $$\:{d}^{2}=2\:{D}_{1}{t}_{\mathrm{m}\mathrm{a}\mathrm{x}}+4{D}_{1}{k}_{\mathrm{o}\mathrm{f}\mathrm{f}}\:{t}_{\mathrm{m}\mathrm{a}\mathrm{x}}^{2}\:$$

For zero *k*_off_ Eq. 3 transforms into Eq. 2. Else, because $\:{t}_{\mathrm{m}\mathrm{a}\mathrm{x}}^{2}$ is positive, *d*^2^ increases faster than linearly with *t*_max_. Fitting Eq. 3 to the data plotted in Fig. 5 yields *D*_1_ ≈ 2150 µm^2^ s^− 1^. The satisfactory quality of the fit rules out the possibility that the microinjection induced significant convection.

**Second**, we fitted the time courses of fluorescence intensity changes using a one-dimensional model (Eq. 1) and a three-dimensional model (Eq. 4):

4 $$\:c\left(x,t\right)={c}_{0}+\frac{A}{(4{\uppi\:}{{D}_{3}t)}^{\frac{3}{2}}}\mathrm{exp}\left(\frac{-{x}^{2}}{4{D}_{3}t}\right)$$

**Fig. 6:**
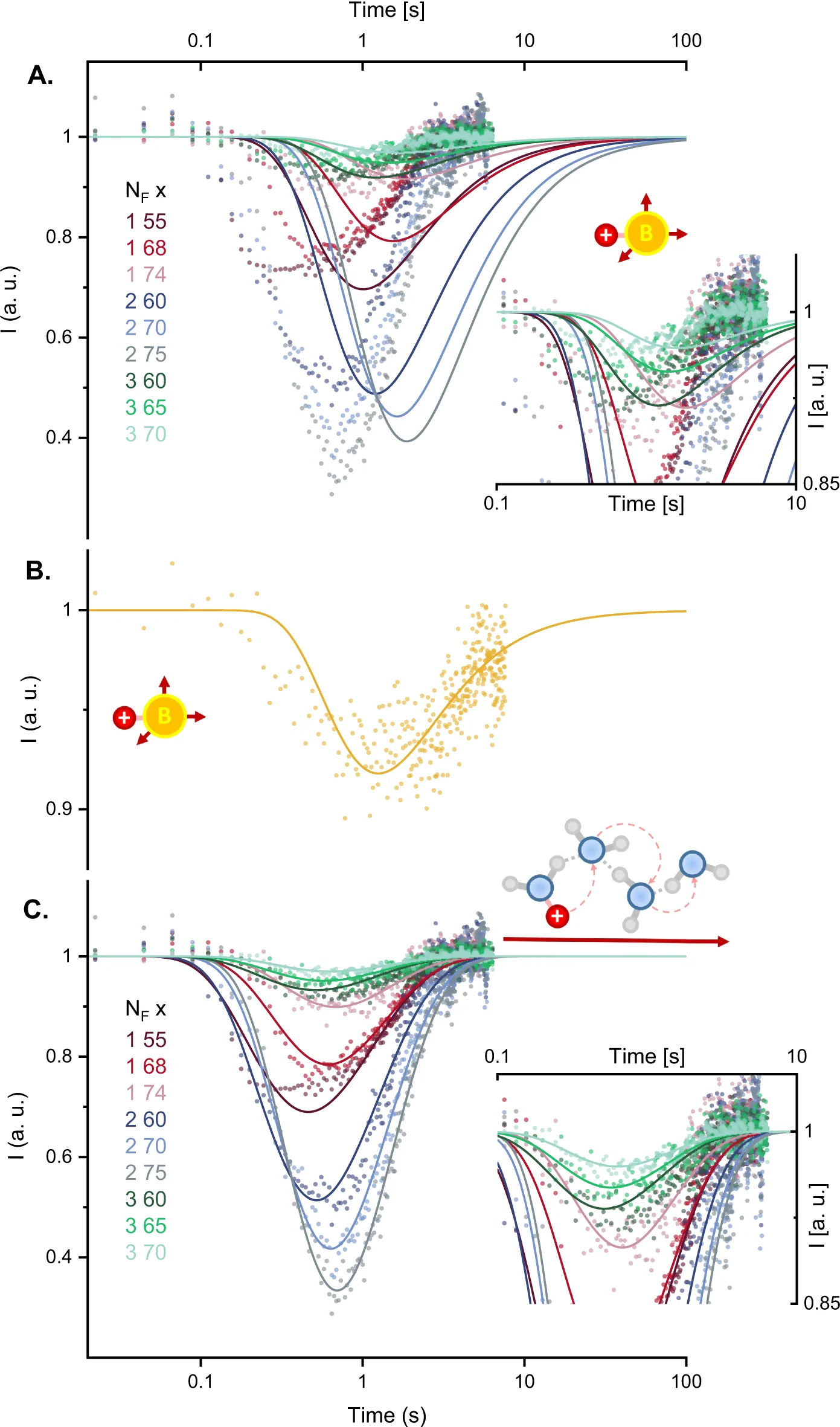
Time-resolved fluorescence changes associated with proton propagation along collagen fibers and comparison with bulk diffusion. Time-resolved fluorescence signals were recorded at nine observation points along three collagen fibers. Fiber 1 is shown in different shades of red, fiber 2 in shades of blue, and fiber 3 in shades of green. Within each color group, darker to lighter shades correspond to increasing distances between the observation points and the proton release site (fiber number *N*_F_ and distances *x* are indicated in the inset). All nine data sets were analyzed by global fitting using a single parameter set. (**A**) Three-dimensional diffusion model assuming buffer-mediated proton transport (Eq. 4), with the diffusion coefficient fixed to that of CAPSO. This model fails to reproduce the measured time courses for proton propagation along the fiber surface. The schematic inset illustrates buffer-mediated transport, with a proton (red) transiently bound to a buffer molecule (yellow). (**B**) Control experiment probing bulk-mediated proton transport. Protons were injected from the bulk solution and fluorescence was recorded at the nearest point on the fiber located 48 μm from the injection site. Under these conditions, the three-dimensional diffusion model (Eq. 4) accurately describes the data, yielding a diffusion coefficient close to that of CAPSO (D_3_ = 307±9 µm^2^s^-1^). The inset depicts the same buffer-mediated transport mechanism as in panel A. (**C**) One-dimensional diffusion model describing proton propagation along the fiber surface (Eq. 1), including an exponential term accounting for proton release into the bulk. This model provides the best overall description of the surface-propagation data. The inset schematically illustrates interfacial proton transport via proton hopping along a hydrogen-bonded water network (Grotthuss mechanism), with the excess proton shown in red, oxygen atoms in blue, and the remaining hydrogen atoms in gray. Because the pH excursions for fiber 3 are comparatively small, the corresponding traces are shown enlarged in a additional insets (in panels **A** and **C**) to allow assessment of the fit quality

Comparing the fit quality of the two models should reveal whether protons primarily moved along the surface of the collagen fibers or within the aqueous bulk. Movement along a collagen fiber can be treated as one-dimensional, since the fiber diameter is negligible compared to the proton travel distance, which reaches tens of micrometers. To increase the robustness of model discrimination, we performed a global fit of all fluorescence time traces shown in Fig. 6A and C. The dataset comprised measurements from three independent collagen fibers, with fluorescence transients recorded at three distinct distances along each fiber. The global fit yielded a single parameter set that simultaneously described all data sets, demonstrating internal consistency across fibers, distances, and repeated measurements.

Although absolute fluorescence amplitudes varied between fibers, these differences primarily reflect variations in the total number of protons delivered during microinjection due to changes in the relative geometry between the microinjection pipette and the fiber, including distance and orientation. Importantly, despite these amplitude differences, the extracted proton diffusion coefficient *D*_1_ and release rate constant *k*_off_ were identical for all fibers within experimental uncertainty. This demonstrates that proton propagation along collagen surfaces is governed by intrinsic interfacial properties rather than by variations in the number of protons delivered or other experimental fluctuations.

The data further indicate that proton propagation occurred much faster than would be permitted by bulk diffusion mediated by the buffer molecule CAPSO (*D*_3_ = 420 µm^2^ s^− 1^). This conclusion follows from the observation that the three-dimensional diffusion model (Eq. 4) failed to reproduce the measured time courses (Fig. 6A). In this model, proton transport in aqueous solution is assumed to occur not via independent diffusion of free protons over macroscopic distances, but predominantly through buffer diffusion. This description is appropriate when the change in free proton concentration Δ[H^+^] induced by microinjection is small compared to the acid equivalents Δ*B* required to drive the observed pH shift, as determined by the buffer capacity *β*.

The following calculation shows that this condition is fulfilled under our experimental settings (*C* = 0.1 mM CAPSO, pK_a_=9.6, initial pH = pH_0_ = 9.0). For a monoprotic buffer, *β* is given by:

$$\begin{aligned}\:\beta\:\left(\mathrm{p}\mathrm{H}\right)&=\frac{\mathrm{d}B}{\mathrm{d}\left(\mathrm{p}\mathrm{H}\right)}\mathbf{}\:\\&=2.3\left(\frac{{C\:K}_{\mathrm{a}}\mathbf{}\left[{\mathrm{H}}^{+}\right]\mathbf{}}{{\left({K}_{\mathrm{a}}\mathbf{}+\left[{\mathrm{H}}^{+}\right]\right)}^{2}}\:+\left[{\mathrm{H}}^{+}\right]+\frac{{K}_{\mathrm{w}}}{\left[{\mathrm{H}}^{+}\right]}\right)\end{aligned}$$

and the acid equivalents per liter required to shift the pH from pH_0_ to a minimum value pH_min_ are

$$\:{\Delta\:}B={\int\:}_{\:{\mathrm{p}\mathrm{H}}_{\mathrm{m}\mathrm{i}\mathrm{n}}}^{{\mathrm{p}\mathrm{H}}_{0}}\beta\:\left(\mathrm{p}\mathrm{H}\right)d\left(\mathrm{p}\mathrm{H}\right)\mathbf{}\mathbf{}\mathbf{}$$.

In contrast, the corresponding change in free proton concentration is:

$$\:{\Delta\:}\left[{\mathrm{H}}^{+}\right]={10}^{-{\mathrm{p}\mathrm{H}}_{\mathrm{m}\mathrm{i}\mathrm{n}}\:}\text{}-{10}^{-{\mathrm{p}\mathrm{H}}_{0}}\text{}$$

For the pH excursions observed here, Δ*B* exceeds Δ[H^+^] by factors of ∼10^2^ – 10^4^. Consequently, the overwhelming majority of the injected protons are transiently buffered, implying that any bulk-mediated propagation would be limited by CAPSO diffusion and therefore too slow to account for the experimental observations.

Importantly, the validity of the three-dimensional bulk diffusion model (Eq. 4) was verified in a dedicated control experiment. A collagen fiber was lifted from the coverslip using the first micropipette, while a second micropipette was used to inject protons into the bulk solution. Fluorescence was recorded at the point on the fiber surface closest to the injection site, at a distance of approximately 48 μm. Under these conditions, the resulting fluorescence time course was well described by Eq. 4, and fitting yielded an effective diffusion coefficient close to the known diffusion coefficient of CAPSO. This control demonstrates that Eq. 4 accurately captures bulk, buffer-mediated proton transport and confirms that its failure in the surface-propagation geometry reflects a genuine change in the dominant transport mechanism rather than a limitation of the model.

Having established that the three-dimensional model (Eq. 4) correctly describes bulk-mediated proton transport (Fig. 6B), but fails to account for proton migration along a collagen fiber (Fig. 6A), we next examined whether a one-dimensional model (Eq. 1) provides an appropriate description. In this framework, proton transport does not rely on buffer molecules or other carriers; instead, protons are assumed to propagate along interfacial water molecules, consistent with proton hopping via the Grotthuss mechanism. Indeed, fits obtained with the one-dimensional model (Fig. 6C) were of substantially higher quality than those obtained with three-dimensional diffusion (Fig. 6A). The fitted diffusion coefficient, *D*₁ = 1776 ± 62 µm^2^s^− 1^, closely matched the value obtained from fitting the proton concentration maxima (Fig. 5).

Taken together, the application of different diffusion models indicates that Eq. 1 best describes the observed proton concentration time courses on collagen fibers. In other words, protons are able to migrate within the interfacial confinement of collagen fibers for tens of micrometers before being released into the surrounding bulk phase.

The global fit of Eq. 1 to the data yielded a proton release constant *k*_off_ of 0.9 ± 0.1 s^− 1^, which compares well to *k*_off_ = 0.5 s^− 1^ from Fig. 5. Within the framework of transition state theory, *k*_off_ allows estimation of Δ*G*^‡^ = 30 *k*_B_T (Weichselbaum et al. 2017):

5 $$\:{k}_{\mathrm{o}\mathrm{f}\mathrm{f}}={\nu\:}_{0\:}\exp\left(\frac{-\varDelta\:{G}^{\ddagger}}{{k}_{\mathrm{B}}\:T}\right)$$

where *T* = 294 K is the absolute temperature, *k*_B_ is the Boltzmann constant, ν_0_ ≈ 10^13^ s^− 1^ is the universal transition state theory attempt frequency for rate processes at surfaces (Barth et al. 2000).

## Discussion

Our data demonstrate that protons can migrate along collagen fibers for tens of micrometers without immediate equilibration with the surrounding bulk solution. This behavior closely parallels lateral proton diffusion observed at membrane–water interfaces (Serowy et al. 2003; Springer et al. 2011), where protons are transiently confined to the surface by an entropic free-energy barrier (Weichselbaum et al. 2017, 2023) associated with interfacial water structuring (Mallick and Agmon 2025; Nguyen et al. 2018; Zhang et al. 2012).

Applying transition state theory to the measurend proton surface diffusion along collagen, yields a Gibbs activation energy barrier Δ*G*^‡^ of approximately 30 *k*_B_T, which is in striking agreement with values reported for lipid membranes (Weichselbaum et al. 2017, 2023). This similarity suggests that proton retention does not arise from binding to specific titratable groups, but rather from confinement within a structured hydrogen-bond network of interfacial water molecules. In this picture, the barrier opposing proton release into the bulk is predominantly entropic in origin.

These findings reinforce the concept that lateral proton conduction on collagen structures depends on a hydrogen-bonded network of water molecules at the surface (Furuseki and Matsuo 2022; Matsui and Matsuo 2020; Orieshyna et al. 2023) much like that found at membrane interfaces.

In other words, protons do not propagate by hopping between titratable residues on the collagen surface; instead, they migrate along interfacial water molecules. Protons bound to titratable residues remain trapped there for times that are far too long to contribute to lateral propagation (Springer et al. 2011). For this reason, local variations in fluorescein density do not affect proton wave propagation (Fig. 4) and can safely be neglected. Models that assume proton diffusion along surfaces via binding to titratable surface groups predict diffusion coefficients that are orders of magnitude too small (Springer et al. 2011).

Most importantly, our experiments confirm that a one-dimensional model of proton diffusion fits the observed propagation dynamics significantly better than three-dimensional diffusion. Thus, protons remain transiently localized along fibers, held by an energy barrier, prior to eventual escape into the bulk.

Studying the magnitude and regulation of this barrier is relevant for a variety of applications. In biotechnology, for instance, retaining protons near interfaces is crucial for proton exchange membranes in fuel cells, which rely on sustained, directed conduction. Collagen-based materials may thus serve as a basis for the design of new, biocompatible proton conductors.

The demonstration that under our experimental conditions protons migrate for tens of micrometers along collagen fibers before escaping into the bulk solution, may be relevant in the context of tumor invasion. If similar behavior persists under physiological buffering conditions and within the complexity of the extracellular matrix, our results suggest a physical mechanism by which aligned collagen networks could focus extracellular acidification toward regions of active matrix remodeling and cell migration. Such localized acidification may enhance the activity of proteolytic enzymes. This process may enhance the efficiency of proteolytic enzymes and ion transporters recruited by migrating cancer cells, thereby promoting invasion through the tumor stroma. The directional flow of protons along aligned collagen networks could thus represent a fundamental pathway coordinating the spatially restricted acidification necessary for matrix digestion and cell motility. Future studies should investigate these relationships in complex tissue environments and determine whether manipulation of collagen alignment or proton conductivity might offer novel strategies for therapeutic targeting of tumor invasion.

## Data Availability

All study data are included in the article and have been deposited in Zenodo at https://doi.org/10.5281/zenodo.21510529.
